# Affective Norms for 718 Polish Short Texts (ANPST): Dataset with Affective Ratings for Valence, Arousal, Dominance, Origin, Subjective Significance and Source Dimensions

**DOI:** 10.3389/fpsyg.2016.01030

**Published:** 2016-07-04

**Authors:** Kamil K. Imbir

**Affiliations:** Faculty of Psychology, University of WarsawWarsaw, Poland

**Keywords:** short texts, affective norms, sentences, polish language, self-assessment manikin

## Abstract

Affective sciences are of burgeoning interest and are attracting more and more research attention. Three components of stimuli meaning have traditionally been distinguished: valence (degree of pleasantness), arousal (degree of intensity of sensations), and dominance (degree of control over sensations). Recently, another three dimensions have been introduced to measure qualities connected to the emotion–duality model: origin (the main component originating in the heart or in the mind), subjective significance (the degree of the subjective goal’s relevance), and source (the location of the stimuli evoking the state). All six affective dimensions were assessed in our study of 718 Polish short texts (sentences of 5–23 words and 36–133 characters in length) describing situations or states in a way that can be referenced to an individual’s experience. Assessments were carried out by 148 psychology students (all women for 108 sentences) and 2,091 students of different faculties (social science, engineering, life science, and science) from Warsaw colleges and universities (1,061 women and 1,030 men for all 718 sentences). Assessing sets of sentences for emotional response is especially useful for researchers interested in emotion elicitation through the use of a phrase such as “imagine that …” or by simply reading emotionally charged material that is more complex and that provides better context than single pictures or words.

## Introduction

Short texts or sentences are a useful method for complex affective process elicitation due to their context information that results from sentence structure (Pinheiro et al., 2016). For example, the word “infection” may be judged as evoking negative feelings, or in some cases treated as a quite neutral medical term. But when “infection” appears as part of a sentence, such as “We don’t even know how easy it is to catch an infection in a crowd,” the word “infection” is exemplified and clarified as an example of infection anxiety. Knowledge of this association benefits us when we are interested in elicitation of a specific state, as for example, the threat of being infected by invisible germs.

To date there have been only a few databases containing research materials in form of short texts (cf. Bradley and Lang, 2007; Pinheiro et al., 2016). The motivation in introducing Affective Norms for Polish Short Texts (ANPST) was to provide short texts and their reliable affective ratings done by Polish participants. Assessments were done for standard dimensions such as valence, arousal, and dominance (c.f. Osgood et al., 1957), as well as a new assessment related to the emotion–duality model (Jarymowicz and Imbir, 2015), namely origin, significance, and source.

The aim of the current study was to check the affective meaning of prepared materials. The study focused on two different populations: (i) psychology students, mostly females, who often participate in psychology experiments, and (ii) a general student population from different faculties and colleges in Warsaw. The dataset of 718 short affective texts and affective ratings in six different dimensions provides a powerful research method for those interested in affective stimuli consequences for mental states and cognitive processing.

## Method

### Participants

Assessments were conducted in three studies. Studies 1a and 1b were performed for a subset of 108 sentences. In Study 1a, assessments were performed by 148 psychology students (women only, aged 19–39: *M* = 21; *SD* = 2.08) and in Study 1b by 322 students (179 women, 143 men) of different faculties (social science, engineering, life science, and science) from Warsaw colleges and universities (aged 18–40: *M* = 22; *SD* = 2.28). Assessments in Study 2 were performed for a subset of 610 sentences by 1,769 students (882 women, 887 men) of different faculties (social science including psychology, engineering, life science, science) from Warsaw colleges and universities (aged 18–47: *M* = 20; *SD* = 1.88). Final assessments from all participants were calculated on the basis of 2,239 filled-in questionnaires. About 124 participants were disqualified from the study having been rejected for not filling in all of the ratings, filling the same rating for more than 10 sentences in a row, or leaving the questionnaire after fewer than 10 min. Participation was voluntary and unpaid. Researchers instructed the participants about the importance of their assessments for future research.

The Maria Grzegorzewska University bioethical committee approved the design of the study and experimental conditions treatments. Participants provided their verbal informed consent to participate in this study. Written consent was not collected due to anonymity assured to participants. Any personal data of participants were not noted during the procedure. This consent procedure was suggested by the Maria Grzegorzewska University bioethical committee.

### Materials

A whole set of 718 Polish emotive sentences were included in assessments in Study 1 (a and b) and in Study 2. Study 1 sentences were (*N* = 108) taken from our previous research (Imbir, 2013; Imbir and Jarymowicz, 2013) and Study 2 sentences were (*N* = 610) collected by a team of 12 judges. Emotive sentences came from literature quotations, movies, newspapers, television programs, humorous stories, and webpages, and consisted of valenced key words from Affective Norms for Polish Words (ANPW: Imbir, 2015). The list of sentences generated focused on a variety of texts covering proverbs, sayings, jokes, quotes, press headings, and even erotic short messages, each trying to elicit the most emotional meaning. The sentences were often modified in an unspecific manner that allowed the participants to relate the content of the sentences to their lives and experiences (see Dataset). This is especially important when taking into account elicited emotions and the nature of the emotional state itself. Most of the emotive situations we focused on were related to goals, plans, or states of possession (Frijda, 2007).

In Studies 1a and 1b, all 108 sentences were randomly arranged into two different orders. On this basis, two main versions of the questionnaire were created. Then each group was divided into six subsets, each containing 18 sentences. In Study 2, all 610 sentences were randomly arranged into two different orders creating two versions of the questionnaire. Each order was divided into 34 subsets, each containing 18 sentences. Each of the sentence subsets was assigned to different scales. This resulted in six different versions of the questionnaire for one order (a total of 12 in both orders) in Studies 1a and 1b, and 34 different versions of the questionnaire for one order (a total of 64 in both orders) in Study 2. Participants assessed all 108 sentences in Studies 1a and 1b or the 108 (=6 × 18) out of 610 sentences in Study 2.

**FIGURE 1:**
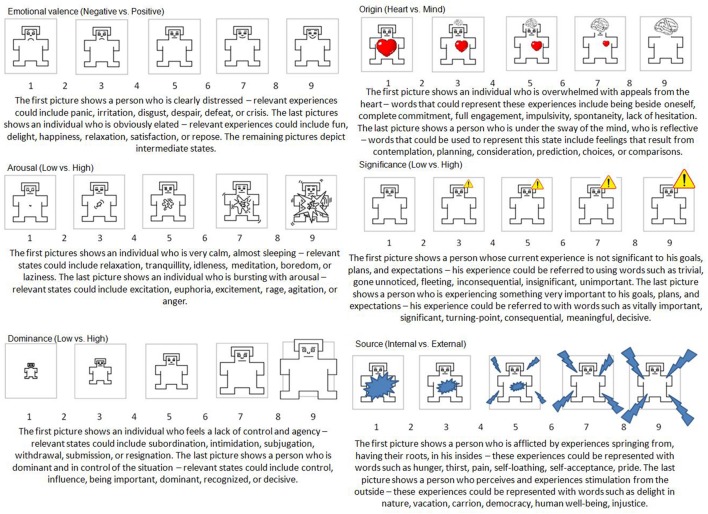
**Self-Assessment Manikin (SAM) scales and their descriptions used in Studies 1a, 1b, and 2 conducted to create Affective Norms for Polish Short Texts (ANPST) dataset**.

To measure the affective meaning of the sentences, six Self-Assessment Manikin (SAM) scales were used, three of which (valence, arousal, and dominance) were adapted from Lang (1980), while the remaining three (origin, significance, and source) were created specifically for research concerning the emotion–duality model (Imbir, 2015). Each SAM scale contained five pictures of people, representing different states, ranging from one state to the opposite state (c.f. Imbir, 2015) and was preceded by a description of the dimensions (c.f. Imbir, 2015). The aim of such detailed descriptions was to minimize ambiguity of the dimensions and to standardize the conditions for each assessment. The descriptions provided an example of each end of the scale of states and feelings to make them more accessible. For example, the origin scale was related to the well-known heart versus mind dichotomy in Polish culture, which was exemplified by a funny television commercial series that has appeared over the past few years. **Figure 1** presents SAM scales and their descriptions used in the current study.

Participants assessed sentences on a 9-point Likert scale where 1 meant negative/calm/being in control/from the heart/of no consequence/internal, and 9 meant positive/excited/controlling/from the mind/important/external. In all SAM scales, 5 was described as a neutral/mixed/moderate state.

### Procedure

For Study 1a, 1b, and Study 2, the procedures were the same. They differed only in the group of participants on which the study was focused (Study 1a vs. Study 1b and 2) or group of assessed sentences (Study 1a and Study 1b vs. Study 2). Each time a group of 108 sentences was assessed by a single participant. All participants assessed sentences using a paper-and-pencil procedure designed as collective sessions in seminar or lecture rooms (from about 20 to 80 participants). Study 1a was conducted in October 2013, Study 1b from March until April 2014, and Study 2 from October until December 2014. All sessions were conducted after regular courses in different departments of Warsaw universities and colleges.

In each session, the researchers explicitly explained the aim of the study to the participants before they started assessing the sentences. The researchers emphasized the importance of the participants’ ratings for the development of research methods, and then they informed the participants about the unpaid and voluntary nature of the participation. The researchers read the meaning of each scale and provided an example. On the assessment sheets, each scale was described and shown on the SAM scale (see **Figure 1**). Then the researchers instructed the participants to ask any questions about the scales before filling in their ratings. The entire procedure lasted no more than 25 min.

There was no time limit for assessments, but participants were encouraged to answer as quickly as possible. The first impression connected with each sentence was the subject of interest in this research. Participants assessed 18 sentences on one of the six different scales (valence, arousal, dominance, origin, significance, and source), then were supposed to change scales to assess the next 18 sentences. Six scales, each accompanied by 18 different sentences, composed the whole questionnaire. The scales’ order was fixed for all questionnaires. This order was designed to move from the easiest then proceed to less intuitive ones (c.f. Imbir, 2015). The participants’ confidentiality was assured, but researchers asked each participant after the assessments to fill in a socio-demographic questionnaire, including gender, age, department, and number of years of education at universities or colleges.

## Results

### Data Treatment

Each sentence in Study 1a was rated by 23–27 participants (*M* = 24.63, *SD* = 1.41) and in Study 1b and Study 2 by 49–56 participants (*M* = 52.27, *SD* = 1.06). The first step was to enter data into the database. Questionnaires (additional *N* = 124) were rejected from participants who resigned from participation in the study. Then descriptive statistics (number of assessments [*N*], Mean [*M*], Standard Deviation [*SD*], and Range) were calculated for each sentence in Study 1 (a and b) and Study 2, separately for each of the six scales. Also, the descriptive statistics for all of the participants’ assessments in both studies were calculated. The supplemental material (Dataset) includes all values, both separate and joint for both studies, for valence, arousal, dominance, origin, significance, and source assessments. All analyses were carried out using IBM SPSS 23 statistical software.

### Descriptive Statistics for Measured Dimensions

In order to describe the results of data collection, the descriptive statistics for the joint assessments of all affective variables used and the lexical dimensions, such as word count, number of letters in the sentence and frequency of appearance were computed. Valence assessments ranged from 1.65 to 8.16 with *M* = 4.74 and *SD* = 1.77. Arousal scale mean assessments ranged from 1.86 to 7.77 with *M* = 4.88 and *SD* = 1.07. Dominance assessments ranged between 2.19 – 7.93 with *M* = 4.69 and *SD* = 1.28. Origin assessments ranged from 1.83 to 8.24 with *M* = 4.64 and *SD* = 1.26. Subjective significance ratings ranged between 1.87 – 7.98 with *M* = 5.25 and *SD* = 1.1. Source scale assessment ranged from 2.41 to 7.40 with *M* = 4.65 and *SD* = 0.85. Taking into account lexical properties of sentences, they varied in length from 5 to 23 words and from 36 to 133 letters, with *M* = 11.69 and *SD* = 3,04 for words and *M* = 77.48 and *SD* = 18.57 for letters. Median frequency of appearance of words composing sentences was ranged from 19 to 1324251 appearances in frequency dataset of Polish texts (Mandera et al., 2014) with *M* = 41183and *SD* = 97084. **Table 1** presents additional analysis including number of sentences and percentage of total number distributed in valence and arousal affective space.

**Table 1:** Summary of sentences distribution over valence and arousal affective space.

		Valence levels			Total
		Negative(<4)	Neutral(4–6)	Positive(>6)	
Arousal levels	Low (<4)	19 (2,6%)	81 (11,3%)	37 (5,2%)	137 (19,1%)
	Medium (4–6)	201 (28%)	125 (17,4%)	146 (20,3%)	472 (65,7%)
	High (>6)	75 (10,4%)	5 (0,7%)	29 (4%)	109 (15,2%)
Total		295 (41,1%)	211 (29,4%)	212 (29,5%)	718 (100%)

*Each dimension divided into 3 categories based on sentence average scores: negative or low arousing: <4; neutral or medium arousing: 4–6 and positive or high arousing: >6.*

### Reliability Estimations

To measure the reliability, split-half estimation based on the version of the questionnaire used was applied (c.f. Imbir, 2015). There were two different orders for the presentation, and each set of descriptive statistics for all sentences was calculated separately. Then both datasets were correlated with the use of Pearson correlation with Spearman–Brown correction due to splitting the whole dataset into two subsamples, thus lowering the number of participants in each. It appeared that all correlations were statistically significant (*p* < .001) and varied from 0.966 for valence, 0.860 for arousal, 0.919 for dominance, 0.899 for origin, 0.855 for significance, and 0.793 for source dimension.

## Discussion

### Possible Usage of ANPST

The developed norms to describe stimuli provide standard research material for researchers interested in emotions and their consequences. These norms are focused on valence, arousal, dominance origin, significance, and source dimensions. Three of these were recently proposed (Jarymowicz and Imbir, 2015) and operationalized in the case of words (Imbir, 2015). The possible usage of a set of 718 words is emotional elicitation by, for example, (i) passive reading or (ii) listening, where a task is only to get to know the material. Another type of possible usage is active processing, using for example (iii) “imagine that ...” procedures, (iv) story writing, or (v) remembering analogical situations in one’s own life. In these types of procedures, sentences are only hints for participants, and participants develop their own affective situations. In such use, norms are useful in preselecting the material, but controlling the evoked state (using proposed SAM scales) is recommended. Sentences, as a type of research material, provide better context of situations and allow for better control of evoked processes by reducing the possibilities of meanings and misinterpretations. Sentences may be used in neuroscience experiments as well, especially focused on text reading, processing, or understanding.

So far, this method has been used to elicit emotions and measure cognitive outcomes of this process in cases of scope of attention (Imbir, 2013) and cognitive control in the anti-saccade test (Imbir and Jarymowicz, 2013). In both experiments, the “imagine that ...” procedure was used with a check of imagined situations properties such as valence and intensity. Both studies showed that sentences could be powerful research methods.

### Data Set Description

The dataset deposited at http://figshare.com/s/e4b4e339138f07c63153 contains an Excel file organized in two ways. First, the short version containing (i) number of participants assessing a single sentence; (ii) mean assessment; and (iii) standard deviation of assessments. In the full version, an additional range is provided for minimum and maximum assessment. In both dataset versions, subsequent columns contain: (i) sentence number; (ii) the sentence in English translation; (iii) the sentence in original Polish; (iv) number of words; and (v) number of letters. Then, mean joint (psychology and non-psychology students) assessment values are given for the six dimensions measured: (vi) valence; (vii) arousal; (viii) dominance; (ix) origin; (x) significance; and (xi) source. The next columns present dimension-by-dimension ratings in detail (*N, M, SD* in short, and *N, Min, Max, M, SD* in full) from Study 1b and 2 (main sample, MS female, and MS male) and from Study 1a (psychology). For example, the short spreadsheet columns from L to W present in detail the results for the valence dimension, and the next 12 columns concern arousal, then dominance, origin, significance, and source. In the full spreadsheet, each dimension covers 20 columns (additional range data). In both versions after affective ratings, additional frequency estimations (calculated mean and median for words constituting a sentence taken from Subtlex-pl (Mandera et al., 2014)) and percentage of parts of speech (nouns, adjectives, verbs, adverbs, conjunctions, prepositions, etc.) included in each sentence are provided (calculated on a basis of Subtlex-pl (Mandera et al., 2014) dataset).

## Author Contributions

The author confirms being the sole contributor of this work and approved it for publication.

## Conflict of Interest Statement

The author declares that the research was conducted in the absence of any commercial or financial relationships that could be construed as a potential conflict of interest.
